# Lymphoedema After Breast Cancer Treatment is Associated With Higher Body Mass Index: A Systematic Review and Meta-Analysis

**DOI:** 10.24248/EAHRJ-D-19-00009

**Published:** 2019-11-29

**Authors:** Astère Manirakiza, Laurent Irakoze, Lin Shui, Sébastien Manirakiza, Louis Ngendahayo

**Affiliations:** a Department of Oncology, Karuzi Fiftieth Hospital, Karuzi, Burundi; b Department of Oncology, University Hospital Centre of Kamenge, Bujumbura, Burundi; c Department of Endocrinology, First Affiliated Hospital, Chongqing Medical University, Chongqing, China; d Department of Oncology, West China Medical Center, Sichuan University, Chengdu, China; e Faculty of Medicine, University of Burundi, Bujumbura, Burundi; f Department of Radiology, University Hospital Centre of Kamenge, Bujumbura, Burundi; g Department of Pathology, University Hospital Centre of Kamenge, Bujumbura, Burundi

## Abstract

**Background:**

Excess body weight has been identified as an important risk factor for lymphoedema following breast cancer treatment, however it remains unclear how much risk increases as weight increases. We conducted a meta-analysis to assess the relationship between body mass index (BMI) and risk of lymphoedema in breast cancer patients, and to estimate the level of risk by BMI category.

**Methods:**

We conducted a systematic search of all articles published through May 2018 in PubMed and the Cochrane library. Studies that included data on BMI and lymphoedema in breast cancer patients were included in the meta-analysis. We compared risk of lymphoedema in BMI groups as: BMI<25 versus BMI≥25, BMI<25 versus BMI≥30, BMI≥25 to <30 versus BMI≥30, BMI<30 versus BMI≥30, BMI<25 versus BMI≥25 to BMI<30.

**Results:**

After exclusion of ineligible studies, 57 studies were included in the meta-analysis. The mean difference in BMI between patients with lymphoedema compared to those without lymphoedema was 1.7 (95% CI, 1.3–2.2). Compared to patients with a BMI<25, risk of lymphoedema was higher in those with a BMI >25 to <30 (odds ratio [OR] 1.3; 95% CI, 1.2 to 1.5), a BMI≥25 (OR 1.7; 95% CI, 1.5 to 1.9), or a BMI≥30 (OR 1.9; 95% CI, 1.6 to 2.4). Compared to patients with a BMI of >25 to <30, risk of lymphoedema was higher in patients with a BMI>30 (OR 1.5; 95% CI,1.4 to 1.8).

**Conclusion:**

Excess body weight is a risk factor for lymphoedema following treatment of breast cancer, with the magnitude of risk increasing across higher categories of BMI.

## INTRODUCTION

Lymphoedema of the upper limb is a complication of breast cancer treatment, especially mastectomy, radiation therapy and chemotherapy.^1^ It results from reduced lymphatic drainage and stasis of fluid in the extremities,^2^ and can occur during treatment or develop years after treatment has been completed.^3^ Estimates of the prevalence of lymphoedema following breast cancer treatment are imprecise due to inconsistencies in the definition of lymphoedema.^4–8^ However, 1 systematic review found that more than 1 in 5 women who survive breast cancer developed lymphoedema.^9^ Several clinical factors have been associated with increased risk of lymphoedema, including: breast surgery, axillary lymph node dissection, sentinel lymph node dissection, radiation therapy, and postoperative infections.^10,11^ Obesity has been identified as the primary demographic factor associated with increased risk of lymphoedema of the upper limb following breast cancer treatment.

A number of studies have examined the relationship between obesity and development of arm lymph-oedema after breast cancer treatment, with the majority finding that. However, most studies do not report on the frequency of lymphoedema within strata of women who are normal weight, overweight or obese, thus precise estimates on the level of risk associated with each weight strata are lacking. Given the high frequency of overweight and obesity among breast cancer patients, clarification of the level of risk of lymphoedema after breast cancer treatment in overweight or obese women is needed to enhance clinical management of breast cancer in this patient subgroup.

We sought to address this knowledge gap by conducting meta-analyses to assess 1) whether body mass index (BMI, defined as weight in kilograms divided by height in metres squared) differs in breast cancer patients with and without lymphoedema after breast cancer treatment, and 2) risk of lymphoedema after breast cancer treatment in subgroups of BMI.

## METHODS

### Search Strategy

A systematic search of all articles published in the English language up to 23 May 2018 was conducted on PubMed and the Cochrane library, using MeSH key words: “breast cancer and lymphoedema”. All references resulting from the MeSH search were imported into Endnote X8, and were examined by 2 independent reviewers. During their first round of review, each reviewer evaluated study titles; those that did not contain the targeted search terms were excluded. During the second round of review the full-text of retained study were evaluated to determine if it was potentially eligible for inclusion in the meta-analyses. Discrepancies between reviewers were resolved via discussion.

### Inclusion and Exclusion Criteria

**FIGURE 1.**
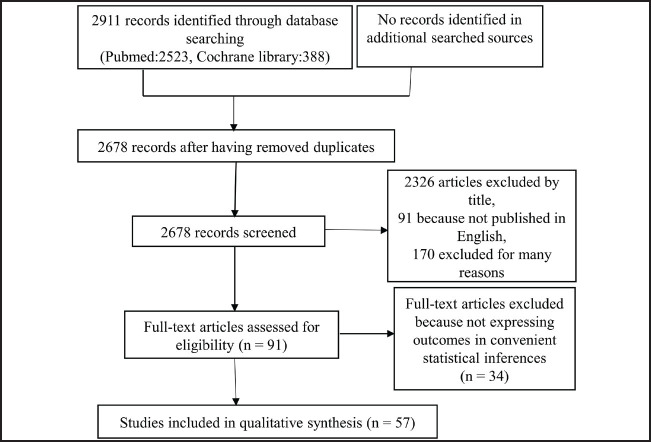
PRISMA Flow Diagram

The inclusion criteria were: publication in English in a peer-reviewed science or medical journal; assessment of BMI (as a continuous or categorical variable) and lymphoedema in female breast cancer patients; and a period of follow-up less than or equal to 10 years. No published abstracts were included. Included and excluded studies are summarised in Figure 1.

### Data Extraction

The following variables were extracted from the published papers (Table): authors, year of publication, study design, patients, type of data, duration of the study, proportion of lymphoedema, lymphoedema evaluation, country where the study was conducted and the definition of lymphoedema. Where available, we extracted patient BMI as a continuous variable; means and ranges were adjusted into mean and standard deviation using the method described by Wan X.^12^ Data on BMI were also extracted as a categorical variable, and where appropriate, regrouped to represent the following categories: BMI<25, BMI≥25, BMI≥25 to <30, and BMI≥30.

**TABLE.** Characteristics of Studies Included in the Analysis

Authors	Year	Design	Patients	Duration	PL	Lymphoedema Evaluation	Country	Lymphoedema Definition
Boughey, J. C.	2014	Prosp.	Patients with breast cancer who underwent unilateral breast conserving surgery	30 months	40.28	Clinical signs of edema and erythema	USA	Presence of clinical impression of breast lymphoedema (BLE) at 2 or more visits beyond 1 month after surgery or a presence of clinical impression of BLE at 1 visit greater than 1 month after surgery with either moderate or severe edema or erythema
Card, A.	2012	Prosp.	Female breast cancer underwent mastectomy	6 years	6.79	Arm circumference measurements	USA	NM
Clark, B.	2005	Prosp.	Women underwent surgery related to breast cancer	18 months	20.7	Arm circumference measurements	UK	Upon measurement, a Percentage Volume Difference change was found to be 5% or more
Crosby, M. A.	2012	Retro.	Breast cancer patients who benefited immediate postmastectomy breast reconstruction	6 years	3,6	Subjective or objective data in medical records	USA	NM
Dominick, S. A.	2013	Prosp.	Patients with early breast cancer	4 years	29.5	Self-report	USA	Swelling of the arm or hand due to fluid buildup following surgery
Geller, B. M.	2003	Prosp.	Women with breast tumor	2 years	3.2	Self-report	USA	NM
Green, J. M.	2013	Prosp.	Women who had been diagnosed with breast cancer and scheduled for surgery	30 months	64.86	Limb volume measurements by circumferences and Perometer	USA	Change in Limb Volume is 5% or greater than the change in BMI
Hinrichs, C. S.	2004	Retro.	Women treated with Postmastectomy radiotherapy for breast cancer	6 years	27	Clinical	USA	Presence of ipsilateral arm edema noted by a treating physician
Hua-Ping, H.	2012	Prosp.	Postmenopausal breast cancer patients with radical mastectomy	18 months	42.9	Circumferential measurement	China	A difference of ≤2 cm at any level between the affected and unaffected limbs
Jammallo, L. S.	2013	Prosp.	Breast cancer patients without metastasis and treated by unilateral breast surgery	7 years	5	Perometer preoperatively and postoperatively	USA	Relative volume change
Jeffs, E.	2016	C.S	Breast cancer patients who had attended a “reducing your risk of lymphoedema” class	6 years	23	Self-report, Clinical assessment and Perometer measurement	UK	At least 10 % excess limb volume, as measured by the Perometer
Jung, S. Y.	2014	Retro.	Patients with stage II or III breast cancer underwent curative breast surgery	6 years	42.22	Circumference measurement and self-perception of arm edema	Korea	Ipsilateral arm swelling of more than 5 % of the circumferential difference without special conditions to the contralateral arm
Kim, M.	2015	Retro.	Clinically node-positive breast cancer patients who underwent Neoadjuvant chemotherapy followed by modified radical mastectomy or BCS with ALND and radiation therapy	6 years	42	Circumference measurement and patient perception of arm edema	Korea	Difference of 5%–10% in arm measurement or only self-perception of arm swelling with less than a 5% measurement difference
Kim, M.	2016	Retro.	Breast cancer patients who underwent curative breast surgery	6 years	34.5	Circumference measurement and patient perception of arm edema	Korea	Ipsilateral arm swelling of more than 5 % of the circumferential difference without special conditions to the contralateral arm
Kim, M.	2013	Retro.	Patients who underwent primary surgery with ALND excluded those received neoadjuvant therapy followed by surgery	6 years	17	Circumference measurement and patient perception of arm edema	Korea	Ipsilateral arm swelling of more than 5 % of the circumferential difference without special conditions to the contralateral arm
Kwan, M. L.	2010	Prosp.	Patients with primary invasive breast cancer	22 months	13.3	Electronic medical records	USA	Disease codes
Kwan, M. L.	2016	Prosp.	Women newly diagnosed invasive breast cancer	101 moths	11.6	Self-report	USA	Any event self-reported by the participant that was ≥6 months after the breast cancer diagnosis
Lee, S. H.	2012	Prosp.	Breast cancer survivors	2 years	60.42	Arm circumference.	Korea	Increase in arm circumference at any level by 2 cm or more compared to the contralateral side
Mahamaneerat, W. K.	2008	Prosp.	Postoperative unilateral breast cancer survivors	30 months	19.17	Arm circumferences and limb volume using cylinder volume	USA	Limb Volume increase of at least 5% greater than BMI change during at least 1 visit after the postoperative visit
Mak, S. S.	2009	C.C.	Patients who underwent axillary dissection for breast cancer	NM	50	History and arm circumference	China	The contralateral arm circumference at corresponding as reference
Meeske, K. A.	2009	C.C	Patients diagnosed in situ to Stage III-A primary breast cancer	18 months	24	Self-reported	USA	Swelling due to an accumulation of fluid in their arm, not to be confused with swelling that occurs after surgery
Menezes, M. M.	2016	Prosp.	Patients treated by mastectomy with axillary lymphadenectomy	1 year	33	Clinical and arm circumference measurement	Brazil	Difference >200 ml between the volume of the affected limb and the contralateral limb
Monleon, S.	2015	Retro.	Patients diagnosed primary invasive breast cancer and treated by surgically	5 years	33.4	Upper limb circumference measurement	Spain	Difference of 2 cm or more at any circumference point
Morcos, B.	2014	C.S.	Patients with breast carcinoma and treated by surgery at least 6 months prior to accrual	6 years	21.4	Measurement of the mid-arm and forearm circumference	Jordan	Mid-arm or forearm circumference difference between both limbs of ≥2 cm
Norman, S. A.	2010	Prosp.	Patients with histologically confirmed breast cancer	6 years	37.7	Questionnaire and scoring system to assess lymphoedema	USA	Patient's perceived differences in the size of her hands and arms
Ozaslan, C.	2004	Prosp.	Patients treated by modified radical mastectomy with complete axillary dissection	30 months	28	Arm circumference measurement	Turkey	Difference at any level compared with the opposite upper extremity ≥2 cm
Park, J. H.	2008	Prosp.	Women operated on by the same surgeon in each hospital	8 months	24.9	Arm circumference measurement	Korea	Difference of 2 cm or more at any level compared with the opposite upper extremity
Pinto, M.	2013	C.S.	Patients who underwent mastectomy or breast conserving surgery with unilateral ALND	5 months	50	Self-report	Italy	Based on consensus document of the International Society of Lymphology
Rebegea, L.	2015	Prosp.	Patients with breast cancer + any treatment	3 years	5.9	NM	Romania	NM
Ribeiro Pereira, A. C. P.	2017	Prosp.	Women underwent ALND for breast cancer	16 months	13.5	Circumference measurement	Brazil	Difference of 200ml between the arms
Ridner, S. H.	2011	Prosp.	Women newly diagnosed breast cancer survivors	30 months	19.6	Using Perometer	USA	200 ml or 10% increase in arm volume occurring on the side where breast cancer treatment
Soyder, A.	2014	Retro.	Female patients with 1-sided breast cancer who had surgical intervention to the breast and axilla	15 months	6.9	Arm circumferential measurements	Turkey	Difference of more than 2 cm in the measurements made at the 4 regions compared to the healthy side
Stout, N. L.	2011	Prosp.	Women with early-stage unilateral breast cancer	12 months	50	Volume and girth measurement by Perometer + self-report	USA	3% volume increase of the affected limb from the preoperative measurement and with consideration for the contralateral limb
Swenson, K. K.	2009	C.C.	Patients clinically diagnosed of lymphoedema and unilateral axillary surgery for invasive breast cancer	44 months	50	Measure of Arm Symptom Survey	USA	Having patients rate them on a 5-point Likert scale from 1 (no swelling) to 5 (very severe swelling
Togawa, K.	2014	Prosp.	Women with first primary in situ or stage I-III invasive breast cancer	5 years	29	Self-report	USA	Arm on the side of breast cancer swollen because of accumulation of fluid in the arm
Vieira, R. A.	2016	Retro.	Women with breast cancer	3 years	7.2	Medical records	Brazil	Description in the medical records
Wang, L.	2016	Prosp.	Patients who had been diagnosed with breast cancer and underwent ALND	12 months	31.84	Circumferential measurement	China	Difference of 2 cm or more at any level compared with the opposite upper extremity
Wilke, L. G.	2006	Prosp.	Women with clinical stage T1/2N0M0 biopsy-confirmed invasive breast carcinoma	5 years	6.9	Arm circumference measurement	USA	Increase of 2 cm from the preoperative arm measurement when compared with the contralateral arm
Hahamoff, M.	2018	Retro.	Patients newly diagnosed unilateral breast cancer	2 years	8.04	Bioimpedance and circumference measurement	USA	NM
Can, A. G.	2016	Retro.	Women with previous surgery for breast cancer	15 months	40.5	Arm circumference measurements	Tyrkey	≥2 cm difference between the 2 upper extremities in at least 1 level and/or at least a 10% difference between the 2 upper limb volumes
Soran, A.	2006	Retro.	Female with and without lymphoedema	10 years	33.3	The volume of every part of the limb was calculated by the truncated cone formula according to circumferential measurements	USA	Lymphoedema defined by the amount of LE as percentage of the volume of normal arm (>10%)
Leung, G.	2014	C.S	Women with and without lymphoedema	NM	71.4	Bioimpedance spectroscopy (BIS) of lymphoedema measurement	USA	Resistance ratio for the untreated arm/treated arm was >1.139 or >1.066 for those women who had surgery on the dominant or nondominant side, respectively at any of the BIS assessments
Baltzer, H. L.	2017	Retro.	Breast cancer patients who completed cancer treatment and underwent ipsilateral hand surgery and or radiation therapy	5 years	3.8	Limb circumference, limb volume measurement or clinical evaluation	USA	Limb circumference difference of 2cm of difference or volume difference of 200 ml
Johansson, K.	2002	Retro.	Women treated for breast cancer who developed arm lymphoedema without recurrence of malignancy	19 months	19.32	Medical records	Sweden	NM
Showalter, S. L.	2013	Prosp.	Breast cancer survivors who were at risk for developing BCRL or who had stable BCRL	2 years	9	Water volume displacement measures	USA	Interlimb volume of difference of ≥5 % accompanied by a ≥5 % increase in the interlimb difference when compared to the last measurement time point
Iyigun, Z. E.	2018	Prosp.	Patients with early-stage breast cancer	3 years	21.3	Circumference measurements of the hand, arm, and forearm + Bioimpedance	Turkey	A difference in circumference of the 2 arms of >2 cm and, values below or above −10 and +10
Shahpar, H.	2013	Prosp.	Breast cancer patients	1 year	30	Arm circumference measurement	Iran	Circumference difference ≥2 cm in any point
Ikeda, K.	2014	Retro.	Primary breast cancer patients who underwent breast surgery with ALND	24 months	31.58	Circumference arm measurement	Japan	Circumference difference of 2 cm at any level
Kilbreath, S. L.	2013	Prosp.	Women with early breast cancer	12 months	9.1	Circumference measurement and Bioimpedance spectroscopy	Australia	A ratio ≥1.139 for women in whom the surgery was on their dominant side and a ratio ≥1.066 for those in whom the surgery was on the nondominant side
Smoot, B.	2014	C.S.	Unilateral breast cancer patients who underwent surgery	NM	47.37	Bioimpedance spectroscopy + volume of a truncated cone	USA	Low frequency and 200 ml difference between the affected and unaffected limbs
Smoot, B.	2010	C.S.	Women, with and without lymphoedema, who had completed active breast cancer treatment	NM	50.69	Circumferential assessment + Bioimpedance	USA	NM
Francis, W. P.	2006	Prosp.	Newly diagnosed resectable breast cancer patients	6 years	67.7	Arms circumference measurements	USA	Limb volume or circumferential measurement increased by at least 5%
Goldberg, J. I.	2011	Prosp.	Women with clinically node-negative breast cancer underwent SLNB	4 years	3	Arms circumference measurements	USA	Lymphoedema was defined as L>2 cm for either the upper arm or the forearm
Goldberg, J. I.	2010	Prosp.	Women without history of breast cancer or axillary surgery underwent SLNB for clinically node-negative breast cancer	5 years	5	Arms circumference measurements	USA	Lymphoedema was defined as L>2 cm for either the upper arm or the forearm
Mak, S. S.	2008	C.S.	Patients with breast cancer undergone unilateral axillary dissection	17 months	50	Arms Circumference Measurements	Hong Kong	Differences between 2 arm circumferences at any level.
McLaughlin, S. A.	2013	Prosp.	Women with ALND or SLNB	20 months	5 to 6	Arms measurements	USA	Ratio ≥1,10(10% increase in the ipsilateral arm when compared with changes in the contralateral arm)
McLaughlin, S. A.	2008	Prosp.	Women underwent breast cancer surgery with SLNB, without prior axillary surgery, without history of breast cancer, and had baseline bilateral upper-extremity measurements	5 years	5 to 16	Arms circumference measurements	USA	Lymphoedema >2 cm for either location

Abbreviations: PL, proportion of lymphoedema; Prosp., prospective; Retro., retrospective; C.S., Cross-sectional; C.C., Case–Control; BCRL, breast cancer-related lymphoedema; ALND, axillary lymph node dissection; NM, not mentioned; SLNB, sentinel lymph node biopsy

### Quality Assessment of Studies

We performed quality assessment of studies included in this meta-analysis using 2 tools: the Quality Assessment Tool for Observational Cohort and Cross-Sectional Studies, a 14-item inventory; and the Quality Assessment of Case-Control Studies, a 12-item inventory.^13^ If an observational cohort or cross-sectional study had more than 8 positive items, or a case-control study had more than 7 positive items, the study was deemed to be of high quality.

### Statistical Analysis

In studies with continuous data for BMI, we calculated the mean difference and 95% confidence interval of BMI between patients with lymphoedema and those without lymphoedema. We used data on the number of lymphoedema events among patients within each BMI category to calculate odd ratios of the association between BMI category and lymphoedema. A random effects model, with a random intercept for each study, was developed to obtain pooled mean differences and ORs by study type. Study heterogeneity was assessed using the I2 index. I2>50% with *P*<.1 indicated significant heterogeneity among studies.^14^ Subgroup analyses were performed to obtain OR and mean difference estimates according to the study design, notably, prospective, retrospective, cross-sectional and case-control studies. Review Manager version 5.3 (The Nordic Cochrane Centre, The Cochrane Collaboration, Copenhagen) was used for all statistical analyses.

**FIGURE 2.**
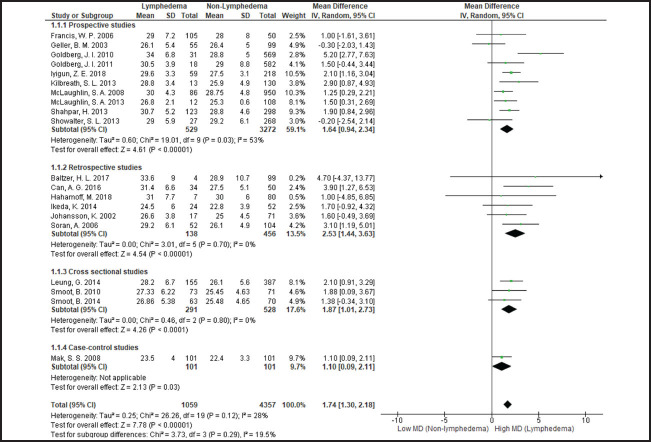
Comparison of BMI Between Patients With and Without Lymphoedema

## RESULTS

### Study Characteristics

We identified 2,911 studies through the MeSH search terms, of which 57 studies, published between 2002 and 2018, were included in the meta-analysis (Figure 1). Among them, 32 were prospective,^15–46^ 15 were retrospective,^47–61^ 7 were cross-sectional^62–68^ and 3 were case-control.^69–71^ The included studies were conducted in USA (30), Korea (6), Turkey (4), China (3), Brazil (3), UK (2),Spain (1), Italy (1), Sweden (1), Romania (1), Japan (1), Australia (1), Hong Kong (1), Iran (1) and Jordan (1). Fourteen studies were classified as low quality and 43 were classified as high quality. The proportion of lymphoedema in prospective studies included in the meta-analysis ranged between 3% and 71.4% (Table). In total, 5,407 participants from 20 studies contributed data for analysis of mean differences in BMI between patients with and without lymphoedema. Overall, 23,208 participants from 38 studies contributed data for analyses of ORs associated with BMI category. Some of the 38 studies did not report on every category of BMI and thus were not included in every OR estimate, while other studies provided data for more than 1 OR estimate. Thus OR estimates of lymphoedema in patients with a BMI<25 versus patients with a BMI≥25 included 33 studies; OR estimates of patients with a BMI<25 versus patients with a BMI≥30 included 20 studies; OR estimates of patients with a BMI≥25 to <30 versus patients with a BMI≥30 included 20 studies; and OR estimates of patients with a BMI<25 versus patients with a BMI≥25 to BMI<30 included 19 studies.

### Differences in BMI between Patients With and With out Lymphoedema

In meta-analysis of 20 studies the overall mean difference in BMI between breast cancer patients with and without lymph-oedema was 1.7 (95% confidence interval [CI], 1.3 to 2.2); heterogeneity among studies was nonsignificant (I2=28%). In subgroup analysis by study design, in all study subgroups BMI was higher in breast cancer patients with lymphoedema compared to those without. However, the mean difference in BMI was higher in retrospective studies 2.5 (95% CI, 1.4 to 3.6), compared to prospective 1.6 (95% CI, 0.9 to 2.3), cross-sectional 1.9 (95% CI, 1.0 to 2.7) and case-control studies 1.1 (95% CI, 0.1 to 2.1). Heterogeneity in prospective studies was moderate (I2=53%) and nonsignificant (I2=0%) in retrospective and cross-sectional studies.

### Odds of Lymphoedema by BMI Category

Breast cancer patients with a BMI in the overweight or obese range more frequently developed lymphoedema than those with a BMI<25, with risk rising across higher BMI categories. Compared to patients with a BMI<25, risk of lymphoedema was higher in those with a BMI in range of 25 to less than 30 (odds ratio [OR] 1.3; 95% CI, 1.2 to 1.5), a BMI≥25 (OR 1.7; 95% CI, 1.5 to 1.9), or a BMI≥30 (OR 1.9; 95% CI, 1.6 to 2.4). Even among overweight or obese patients, higher BMI was associated with a greater frequency of lymphoedema. Compared to patients with a BMI between 25 and less than 30, odds of lymphoedema was 50% higher patients with a BMI≥30 (OR 1.5; 95% CI, 1.4 to 1.8).

Heterogeneity of OR estimates across studies was moderate in overall analyses comparing patients with BMI<25 to those with BMI≥25 (I2=53%) and comparing patients with BMI<25 to those with BMI>30 (I2=49%). Cross-study heterogenity was also moderate in subgroup analysis of prospective studies comparing patients with BMI<25 to those with BMI≥25 (I2=53%) and comparing patients with BMI<25 to those with BMI≥30 (I2=49%). Study heterogeneity was only substantial in subgroup analysis of cross-sectional studies comparing patients with BMI between 25 and 30 to those with BMI≥30 (I2=75%). In all other analyses heterogeneity was nonsignificant.

In subgroup analyses based on study design comparing patients with a BMI<25 to patients with a BMI≥25, mean ORs were higher in cross-sectional studies (OR 2.9; 95% CI, 1.7 to 5.3) and case-control studies (OR 2.4; 95% CI, 1.6 to 3.7) compared to prospective studies (OR 1.7; 95% CI, 1.5 to 2.1), and retrospective studies (OR 1.3; 95% CI, 1.1 to 1.5). In contrast, in subgroup analyses comparing patients with a BMI of 25 to less than 30 to patients with a BMI≥30, the mean OR was higher in prospective studies (OR 1.6; 95% CI, 1.4 to 1.8) compared to retrospective studies (OR 1.3; 95% CI, 0.8 to 1.9) and cross-sectional studies (OR 1.2; 95% CI, 0.1 to 13.6). In subgroup analyses comparing patients with a BMI<25 to patients with a BMI≥30, mean ORs by study types ranged from 1.9 to 2.5 in cross-prospective, cross-sectional and case control studies, with an overall OR of near 2 (OR 1.9; 95% CI, 1.6 to 2.4) (Figure 4). A wider range of mean ORs was observed in subgroup analyses comparing patients with a BMI<25 to patients with a BMI≥25 and less than 30. The OR was lowest in the retrospective study subgroup, which represented a single study (OR 1; 95% CI, 0.6 to 1.7), moderate in the prospective study subgroup (OR 1.3; 95% CI, 1.2 to 1.5), and highest in the cross-sectional study subgroup (OR 1.9; 95% CI, 0.7 to 5.5) and the case-control study subgroup (OR 1.9; 95% CI, 1.2 to 3.2), which also represented a single study (Figure 6).

**FIGURE 3.**
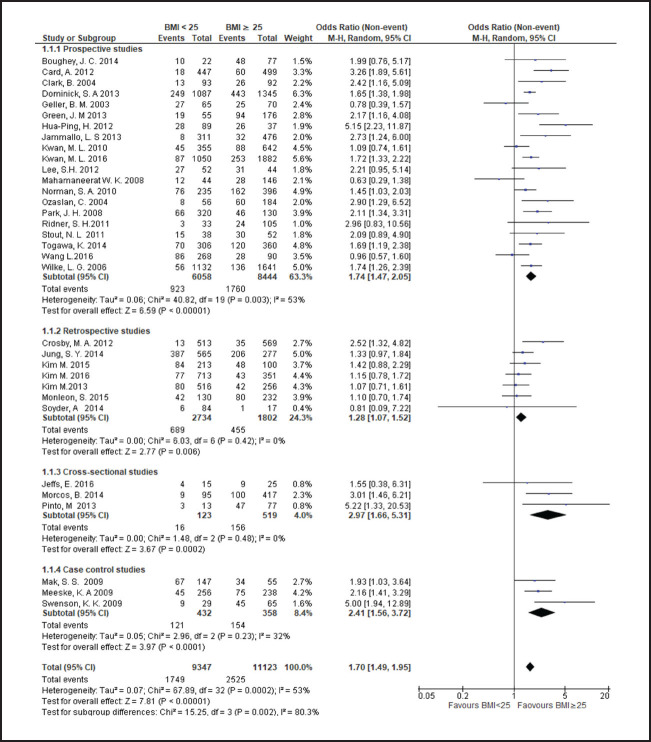
Forest Plot Comparing Lymphoedema in Patients With BMI <25 and Those With BMI ≥25

**FIGURE 4.**
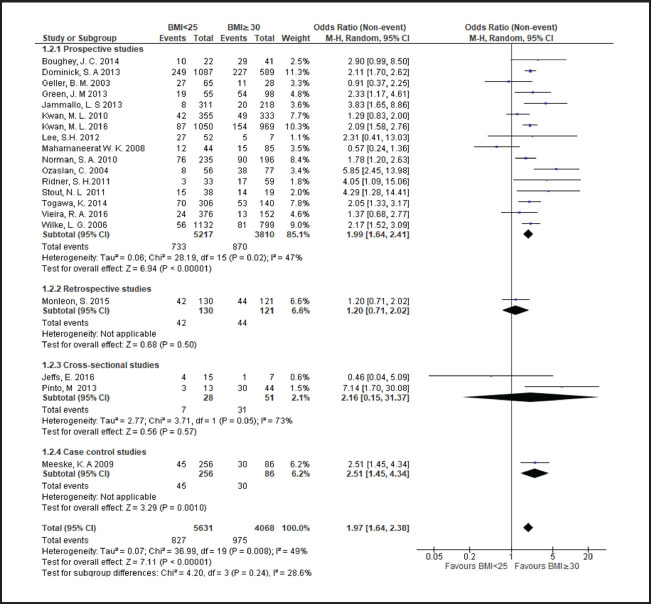
Forest Plot Comparing Lymphoedema in Patients With BMI <25 and Those With BMI ≥30

**FIGURE 5.**
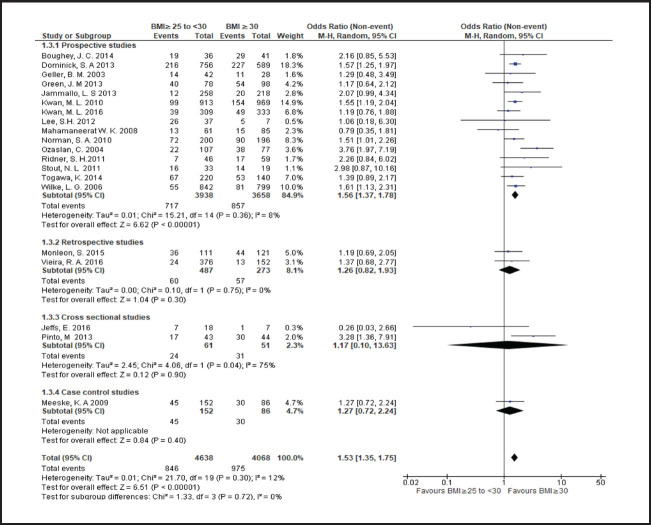
Forest Plot Comparing Lymphoedema in Patients With 25≤BMI<30 and Those With BMI ≥30

**FIGURE 6.**
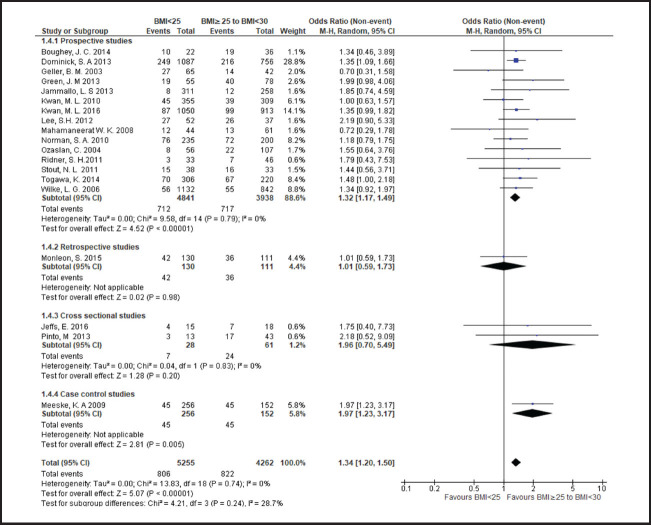
Forest Plot Comparing Lymphoedema in Patients With BMI<25 and Those With 25≤BMI<30

## DISCUSSION

In this meta-analysis, we found strong associations between BMI and lymphoedema in female breast cancer patients. Mean differences in BMI were significantly elevated in lymphoedema patients compared with those who did not develop lymphoedema. Further, compared to a reference BMI value of <25 (that is, at or below normal weight), ORs for lymphoedema increased in magnitude across higher categories of BMI, within the range of 1.3 to 1.9. This finding reflects a trend of increasing risk of lymphoedema with increasing weight reported in individual studies included in this meta-analysis. While ORs of the association of BMI category and lymphoedema from individual studies ranged from 0.3^62^ to 7.1,^64^ only 5 studies reported an OR below 1, reflecting the robustness of our overall estimate. Further, study heterogeneity was moderate to nonsignificant in most analyses and 75% of included studies were of high quality. We observed some variability in the magnitude of ORs by subgroup of study design type, however, subgroup ORs were largely consistent with overall ORs.

Strikingly, we found that even among overweight and obese cancer patients, higher BMI increased risk of lymph-oedema. In particular, our analysis estimated that risk of lymphoedema was 50% higher in patients with a BMI>30 compared to those with a BMI in the range of 25 to less than 30. This finding is supported by a recent meta-analysis of BMI and risk of lymphoedema, which reported an 39% increased risk of breast cancer-related lymphoedema in obese patients compared to overweight patients.^81^ However, lymphoedema is more noticeable, and thus potentially more readily diagnosed in patients with a high BMI compared to those with a normal BMI. While the contribution of diagnostic bias to the observed association between higher BMI and increased risk of lymphoedema is unknown, the observed dose-response relationship between excess body weight and increased risk of lymphoedema suggests a biological link between the 2.

In prospective studies that were included in this meta-analysis, we found a high proportion of lymphoedema, ranging from 3% to 67.7%, with a mean of 24.19%. A similarly high proportion of lymphoedema has been reported in other studies. Based on insurance claim data, 10% of patients had lymphoedema within 2 years of treatment of newly diagnosed breast cancer.^85^ A prospective cohort study of breast cancer survivors reported that within 5 years of treatment 43% to 94% of patients had lymphoedema, with estimates varying depending upon how the lymphoedema was defined.^86^ These incidence estimates are derived from overall patient populations, and may be even higher in subgroups of overweight and obese women in whom risk of lymphoedema is elevated.

The process through which higher BMI may lead to the development of lymphoedema remains unclear but several mechanisms have been proposed. In particular, lipid accumulation throughout the body may impede lymphatic transport of fluids, in a process driven in part by chronic inflammatory responses.^82^ In a mouse model, lymphoedema in obese mice was found to impair lymphatic function, associated with increased subcutaneous adipose deposition, a higher frequency of CD45+ and CD4+ inflammatory cells, and fibrosis without any change in the number of lymphatic vessels.^83^

### Limitations

**FIGURE 7.**
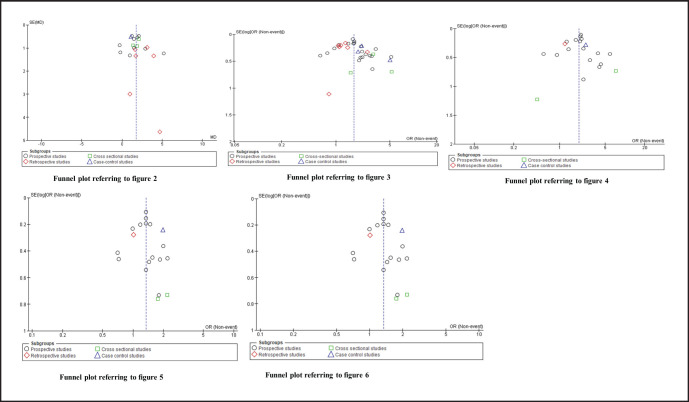
Funnel Plots Referring to Previous Figures

Our meta-analysis has some limitations, which should be considered. Firstly, methods used to diagnose lymphoedema were not consistent across the studies included in this meta-analysis, and some studies did report on how diagnosis was conducted. In some studies, BMI was not a primary variable of interest, and thus may not have been carefully recorded. Further, a variety of study populations were represented across studies, including. While this may improve the overall generalizability of our findings, it may also have resulted in wider confidence intervals around our pooled estimates. Our study did not include 191 non-English citations identified by our MeSH search, which could contain important data not considered in this study. Moreover, the majority of studies included in our meta-analysis were conducted in the USA (52.3%) or in Europe, thus our results may not reflect the impact of BMI on risk of lymphoedema in geographic areas not included in the analysis. The publication biases assessment has been summarised in Figure 7 by using funnel plots. The significant asymmetry was found in the funnel plots referring to Figure 3. This should be caused by heterogeneity within studies.

## CONCLUSION

This meta-analysis showed that being overweight or obese is an important risk factor for developing lymphoedema of the upper limb following breast cancer treatment. Lymphoedema is more noticeable, and thus potentially more readily diagnosed in patients with a high BMI compared to those with a normal BMI. However, our finding that the magnitude of risk of lymphoedeoma rises across higher categories of BMI supports a biological link between being overweight and developing lymphoedeoma. To further clarify the relationship between excess body weight and risk of lymphoedema, future studies should detail methods used to diagnose lymph-oedema and report the frequency of lymphoedema in BMI subgroups from patient populations representing a range of BMI levels.
